# 3D Hierarchical Co–Al Layered Double Hydroxides with Long-Term Stabilities and High Rate Performances in Supercapacitors

**DOI:** 10.1007/s40820-016-0121-5

**Published:** 2016-12-27

**Authors:** Jiantao Zai, Yuanyuan Liu, Xiaomin Li, Zi-feng Ma, Rongrong Qi, Xuefeng Qian

**Affiliations:** Shanghai Electrochemical Energy Devices Research Center, School of Chemistry and Chemical Engineering and State Key Laboratory of Metal Matrix Composites, Shanghai Jiao Tong University, Shanghai, 200240 People’s Republic of China

**Keywords:** Co–Al layered double hydroxides (Co–Al-LDHs), Nanosheets, 3D hierarchical architectures, Butyl alcohol, Supercapacitors

## Abstract

Three-dimensional (3D) flower-like Co–Al layered double hydroxide (Co–Al-LDH) architectures composed of atomically thin nanosheets were successfully synthesized via a hydrothermal method in a mixed solvent of water and butyl alcohol. Owing to the unique hierarchical structure and modification by butyl alcohol, the electrochemical stability and the charge/mass transport of the Co–Al-LDHs was improved. When used in supercapacitors, the obtained Co–Al-LDHs deliver a high specific capacitance of 838 F g^−1^ at a current density of 1 A g^−1^ and excellent rate performance (753 F g^−1^ at 30 A g^−1^ and 677 F g^−1^ at 100 A g^−1^), as well as excellent cycling stability with 95% retention of the initial capacitance even after 20,000 cycles at a current density of 5 A g^−1^. This work provides a promising alternative strategy to enhance the electrochemical properties of supercapacitors.

**Electronic supplementary material:**

The online version of this article (doi:10.1007/s40820-016-0121-5) contains supplementary material, which is available to authorized users.

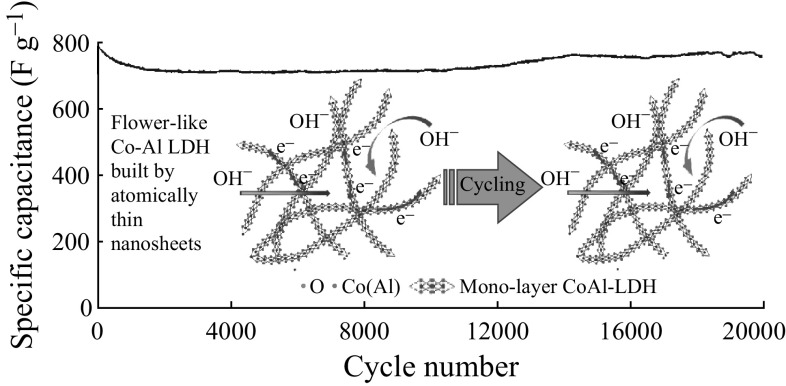

## Highlights

- 3D Flower-like Co–Al layered double hydroxides (Co–Al-LDHs) built up of atomically thin nanosheets were successfully synthesized via a hydrothermal method in a mixed solvent of water and butyl alcohol.
- Owing to the unique hierarchical structure and modification by butyl alcohol, the electrochemical stability and the charge/mass transport of the Co–Al-LDHs was improved, therefore leading to high specific capacitance, excellent rate performance and good cycling stability in supercapacitors.

## Introduction

To meet the increasing demand for clean energy technologies, many energy storage and conversion devices, such as fuel cells, batteries, and supercapacitors, have been developed [1–5]. Compared with other chemical energy storage devices, supercapacitors have attracted extensive attention owing to their fast charge/discharge rate, high power density, and long cycle lifetime [6–10]. Up to now, carbon-based capacitors have been widely studied due to their cost-effectiveness and excellent rate and cyclic stability [6]. However, the relatively low capacitance (<300 F g^−1^) cannot meet the demand for high energy density.

It has been reported that pseudocapacitive transition metal oxides/hydroxides possess high capacitances derived from their reversible faradic reactions [11–14]. Layered double hydroxides (LDHs), which are made up of positively charged brucite-like layers with an interlayer region containing charge compensating anions and solvation molecules, are promising electrode materials for supercapacitors due to the synergistic effects of bi-metal cations, such as reciprocal activation [15, 16]. However, the migration of metal cations can be limited by other cations, which can suppress the aggregation and growth of the active materials [17, 18]. Co–Al-LDHs with divalent Co^2+^ ions and trivalent Al^3+^ ions are one of the most commonly studied LDHs because of their excellent electrochemical properties [19–21]. However, the specific capacitance, rate capability, and stability are usually poor because of the limited conductivity and the re-stacking of 2D nanosheets [22, 23]. Compositing with highly conductive substrates, such as Ni foil or carbon materials, is considered an effective method to improve the performance of Co–Al-LDHs. For example, the porous Co–Al-LDHs/GO (GO, graphene oxide) nanocomposite exhibits a specific capacitance of 1043 F g^−1^ at 1 A g^−1^ [24]. H-OH intercalated Co–Al-LDHs on Ni foil shows a capacitance of 1031 F g^−1^ at 1 A g^−1^ and an ultrahigh rate capability with 66% capability retention at 100 A g^−1^ [25]. However, the cycling stability of LDHs is usually less than 5000 cycles (Table S1), which is far from the practical demand of 100,000–200,000 cycles. Therefore, the stability of Co–Al-LDHs is the most prominent problem to overcome.

In general, active materials for electrodes with larger surface areas show higher capacitances and stabilities. Two-dimensional (2D) monolayer LDH nanosheets with extremely large surface areas can be prepared by a top-down method, in which LDH nanoplates are first prepared and then exfoliated in liquid medium by ultrasonic treatment [26]. However, the nanosheets prefer to re-stack to reduce the surface free energy, which is detrimental to the capacitance and stability of the electrodes. It has been accepted that three-dimensional (3D) hierarchical structures composed of 2D nanosheets are more stable than 2D nanosheets [27, 28]. The unique structure is beneficial to charge and mass transport and the mitigation of volume change during the charge/discharge process [29]. Furthermore, 3D hierarchical structures can supply more points to connect the conductive matrix in the electrodes, which can provide more electron paths and suppress the separation of active materials [30–32]. On the other hand, the stability of the layered compounds can be improved by modification with organic compounds because they can intercalate and/or adsorb into the layers to reduce the surface energy [33–36] and further prevent the re-stacking of nanosheets [37]. For example, Xiao et al. found that MoS_2_/PEO [poly(ethylene oxide)] nanocomposites had high reversible capacities with long-term reversibility because the incorporation of PEO can stabilize the disordered structure of MoS_2_ [38].

Herein, 3D hierarchical Co–Al-LDHs were fabricated in a rationally designed reaction system. Owing to the unique hierarchical structures composed of atomically thin nanosheets and the modification by butyl alcohol, the electrochemical stability and the charge/mass transport of the 3D Co–Al-LDH architectures were improved. When used in supercapacitors, high specific capacitance and good cycling stability were achieved.

## Experimental Section

### Synthesis of 3D Hierarchical Co–Al-LDHs

In a typical procedure, Co(NO_3_)_2_·6H_2_O (2.4 mmol, 0.698 g) and Al(NO_3_)_3_·9H_2_O (0.8 mmol, 0.3 g) were dissolved in 40 mL deionized water and 40 mL butyl alcohol and stirred for 30 min. Then, 0.384 g of urea and 15 mg of citric acid trisodium salt dehydrate were added and further stirred for another 30 min. Next, the mixtures were sealed in a 100-mL Teflon-lined steel autoclave and hydrothermally treated at 120 °C for 12 h. After being cooled to room temperature naturally, the samples were filtered and washed with deionized water and ethanol several times and then freeze-dried (5 × 10^−2^ mbar at *T* ≤ −46 °C) for 24 h to obtain the 3D Co–Al-LDHs. For comparison, 2D Co–Al-LDHs were prepared using deionized water as the solvent, and zero-dimensional (0D) Co–Al-LDHs were prepared using butyl alcohol as the solvent under similar reaction conditions.

### Material Characterization

The crystal structure and phase were characterized on an X-ray powder diffractometer (XRD, Shimadzu-6000) and X-ray photoelectron spectrometer (XPS, VG Scientific ESCLAB 220iXL). The size and morphology of the as-synthesized products were determined by a transmission electron microscope (TEM, JEOL-1200) and field emission scanning electron microscope (FESEM, JEOL, JSM-7401F) with an accelerating voltage of 5 kV. Atomic force microscopy (AFM) measurements were collected on a Multimode atomic force microscope (Veeco Instruments, Inc.). Typically, a freshly diluted ethanol solution of the NiFe-LDH samples was ultrasonically treated and then deposited onto a clean mica wafer by drop-casting. The nitrogen adsorption–desorption measurement was conducted on a Micromeritics ASAP 2010 analyzer, and the specific surface areas of samples were determined by Brunauer–Emmett–Teller (BET) analysis. FT-IR spectra were recorded on a PerkinElmer Spectrum 100 Fourier transform infrared spectrometer using KBr pellets.

### Electrochemical Measurements

The electrochemical experiments were performed using a standard three-electrode configuration with the as-synthesized sample electrode as the working electrode, platinum as the counter electrode, and Hg/HgO as the reference electrode. The electrolyte was a 2 mol L^−1^ aqueous KOH solution. The working electrodes were prepared as follows: 75 wt% active materials were mixed with 7.5 wt% acetylene black, 7.5 wt% KS-6, and 10 wt% polyvinylidene fluoride in NMP. The slurry was pressed on Ni foam (2 cm ×1 cm × 1 mm) and dried at 80 °C under vacuum for 6 h. Each working electrode contained approximately 1 mg of active material. CV and galvanostatic charge/discharge tests were performed on an electrochemical workstation (Zahner Zennium CIMPS-1, Germany) in the potential range of 0–0.55 V and 0–0.45 V, respectively. Electrochemical impedance spectroscopy (EIS) was carried out by applying a 5 mV amplitude over a frequency range of 0.01 Hz to 100 kHz at open circuit potential.

## Results and Discussion

**Fig. 1:**
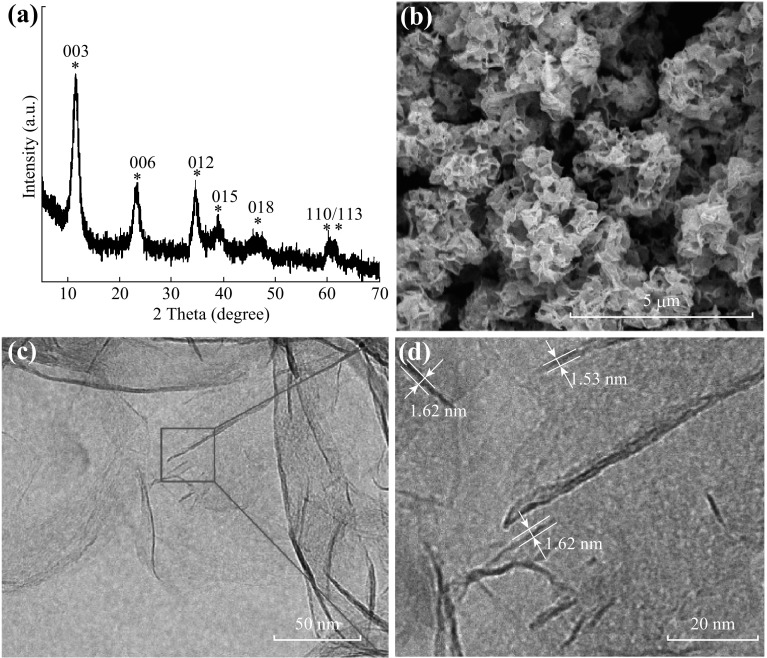
**a** XRD pattern, **b** SEM image, **c** TEM image, and **d** HRTEM image of the as-prepared 3D Co–Al-LDHs

The crystal structure of the 3D Co–Al-LDHs calculated by the XRD pattern are shown in Fig. 1a. The diffraction peaks located at 11.3°, 22.8°, 35.0°, 39.3°, 46.4°, 61.1°, and 62.4° correspond to the (003), (006), (012), (015), (018), (110), and (113) facets, respectively, implying the obtained LDH product has a rhombohedral structure [39]. The XRD patterns (Fig. S1a, b) of the obtained 2D Co–Al-LDHs and 0D Co–Al-LDHs show similar structures. SEM images (Figs. 1b, S2a, b) clearly show the 3D hierarchical structure built up of nanosheets. TEM and HRTEM images (Fig. 1d) further reveal the ultrathin nature with a thickness of approximately 1.6 nm, which can also be confirmed by AFM measurements (Fig. S2c, d). 2D nanosheets with a thickness of approximately 2.5 nm (2D Co–Al-LDHs) and nanospheres with an overall size of approximately 50 nm (0D Co–Al-LDHs) were formed when the mixed solvent was replaced by deionized water or butyl alcohol, respectively (Fig. S1c–f). To evaluate the formation of 3D Co–Al-LDHs, the XRD patterns and SEM images of the products prepared at different reaction times are shown in Fig. S3. The 2D Co–Al-LDH nanoplates were formed when the reaction time was 2 h, and they gradually changed into self-assembled 3D Co–Al-LDHs built up of nanosheets with increasing reaction time. The morphological evolution from the 2D LDH to the 3D LDH nanostructure follows co-precipitation, dissolution, and recrystallization processes. Furthermore, the selective adsorption of butyl alcohol on the {001} facets of LDH can minimize the surface energy to form and stabilize the atomically thin LDH nanosheets [33–36]. The interactions of butyl alcohol adsorbed on the surface of the LDH nanosheets can also help form 3D microspheres by the self-assembly of atomically thin (mono-/bi-layers) LDH nanosheets.

**Fig. 2:**
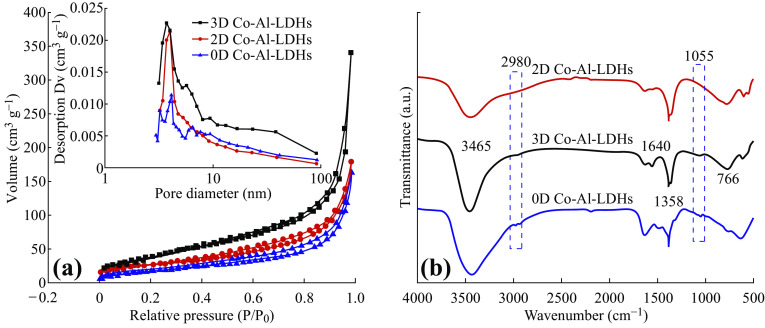
**a** Nitrogen adsorption and desorption isotherms, and **b** FT-IR spectra of all Co–Al-LDHs samples, where the *inset* corresponds to the BJH pore size distribution of all Co–Al-LDH samples

N_2_ adsorption–desorption isotherms of 3D Co–Al-LDHs, 2D Co–Al-LDHs, and 0D Co–Al-LDHs are shown in Fig. 2a. The mesoporous size of 3D Co–Al-LDHs is in the range of 3–10 nm (inset in Fig. 2a). Moreover, the specific surface area of the 3D Co–Al-LDH hierarchical structure is 152 m^2^ g^−1^, and the total pore volume is 0.52 cm^3^ g^−1^, which are much higher than those of the other samples (Table S2) and previously reported results [40–42]. Figure 2b depicts the FT-IR spectra of all samples. The broad adsorption peak at 3465 cm^−1^ is attributed to O–H stretching modes of interlayer water molecules and H-bonded OH groups. The weak peak at 1640 cm^−1^ corresponds to the bending mode of water molecules. The strong peaks at 1358 and 766 cm^−1^ belong to the *ν*_3_ vibrational and bending modes of CO_3_^2−^, respectively [43]. The weak absorption peaks in the range of 800–500 cm^−1^ correspond to the lattice vibrations of the M–O and O–M–O (where M = Co, Al) groups [44]. The faint peaks at 2980 and 1055 cm^−1^ (Fig. S4) in the spectra of 3D Co–Al-LDHs and 0D Co–Al-LDHs belong to C–H and C–C or alkoxy groups (blue dashed line in Fig. 2b), indicating the presence of organic molecules (butyl alcohol) in the samples prepared in mixed solvent or butyl alcohol. No such peaks are detected in the spectrum of 2D Co–Al-LDHs prepared in water, further supporting the existence of butyl alcohol in 3D Co–Al-LDHs and 0D Co–Al-LDHs.

**Fig. 3:**
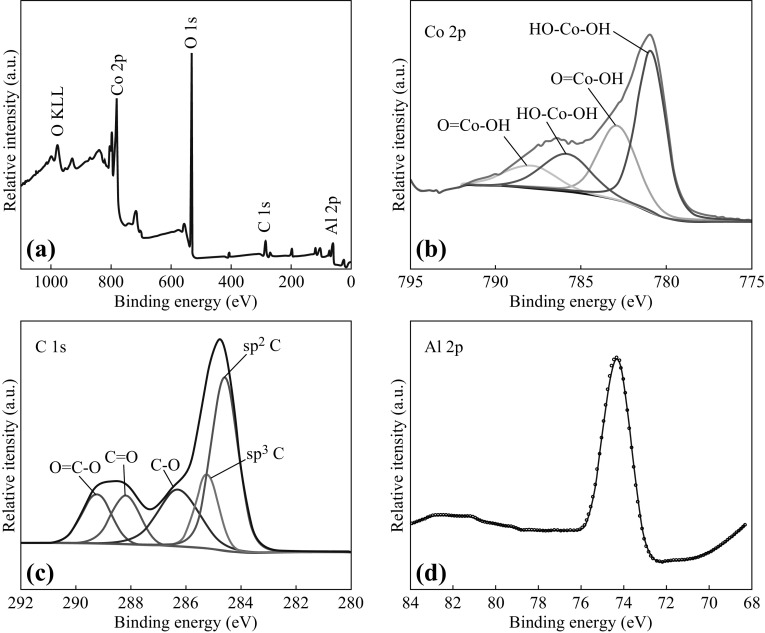
**a** XPS spectrum of 3D Co–Al-LDHs. High-resolution spectra of **b** Co 2*p*, **c** C 1*s,* and **d** Al 2*p*

The XPS spectrum of 3D Co–Al-LDHs shown in Fig. 3a indicates the presence of Co, Al, O, and C. The high-resolution XPS spectrum of Co (Fig. 3b) displays the spin–orbit splitting of Co 2*p* into Co 2*p* ^1/2^ (797.2 and 803.1 eV) and Co 2*p* ^3/2^ (780.9 and 786.6 eV), suggesting the coexistence of Co^2+^ and Co^3+^ [45]. Additionally, the C 1 s peak can be separated into to five peaks centered at 284.6, 285.3, 286.4, 288.2, and 289.4 eV, which may be attributed to *sp* ^2^ C, *sp* ^3^ C, C–O, C=O, and O=C–O, respectively, derived from the adsorbed organic molecules and CO_3_^2−^ groups (see Fig. 3c). The peak at 74.2 eV in the fine spectrum of Al 2*p* is related to the Al^3+^ species in the form of Al–OH [46].

**Fig. 4:**
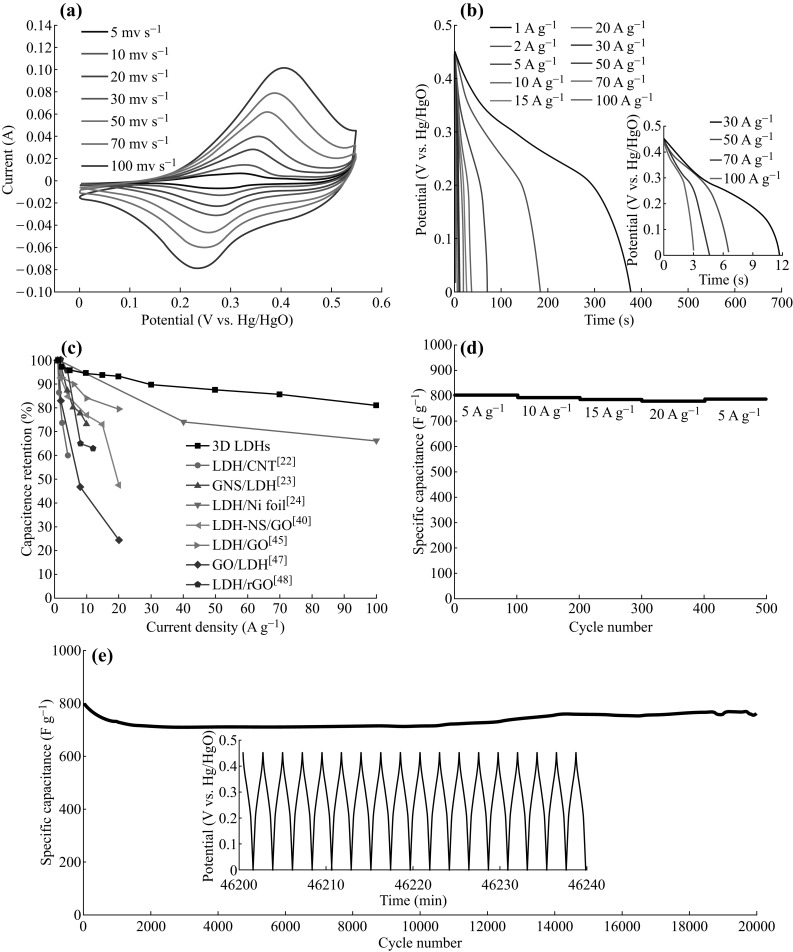
**a** CV curves at different scan rates in 2 M KOH aqueous electrolyte. **b** Galvanostatic charge–discharge measurements at different current densities. Specific capacitances at **c** different current densities, **d** rate performances, and **e** long-term cycling performances of 3D Co–Al-LDHs (LDH: Co–Al-LDH, *CNT* carbon nanotubes, *GNS* graphene nanosheets, *NS* nanosheets, *GO* graphene oxide, *rGO* reduced graphene oxide)

The electrochemical energy storage performances of the obtained samples were studied by a three-electrode cell in the potential range of 0–0.55 V with 2 M KOH aqueous solution as the electrolyte. The specific capacitance of an electrode can be calculated using the following Eq. 1:

1 $$C_{\text{SP}} = {{I \times t} \mathord{\left/ {\vphantom {{I \times t} {(\Delta V \times m)}}} \right. \kern-0pt} {(\Delta V \times m)}},$$

where *I*, *t*, Δ*V,* and *m* stand for the constant current density (A g^−1^), the discharge time (s), the potential (V), and the mass of the electroactive material, respectively. Cyclic voltammetry (CV) curves at various scan rates are shown in Fig. 4a. The symmetrical oxidation–reduction peaks at different scan rates imply the high electrochemical reversibility of 3D Co–Al-LDHs. The specific capacitances of the 3D-Co–Al-LDHs are 838, 813, 801, 792, 783, 780, 753, 732, 715, and 677 F g^−1^ at 1, 2, 5, 10, 15, 20, 30, 50, 70, and 100 A g^−1^ (see Fig. 4b), which are higher than the values of the most often reported carbon-based Co–Al-LDH composites [23–25, 41, 47–49] (Fig. 4c; Table S1). The improved reversibility and rate capability of the 3D Co-Al-LDHs are derived from its abundant active sites and pores in the 3D hierarchical structure, which can further provide accessible pathways for electrolyte and facilitate the transport of ions from the liquid to the LDH. As shown in Fig. 4d, the 3D Co–Al-LDH electrode has a constant capacitance of 801 F g^−1^ in the initial 100 cycles at 5 A g^−1^, and maintains stable retentions of 99, 98, and 97% after every 100 cycles at 10, 15, and 20 A g^−1^, respectively. Furthermore, the 3D Co-Al-LDH electrode also exhibits long-term cycling stability and can still retain approximately 95% of the initial capacitance even after 20,000 cycles (Fig. 4e). The similar potential response of each charge–discharge curve also indicates the high reversibility of the charge–discharge process (inset in Fig. 4e).

**Fig. 5:**
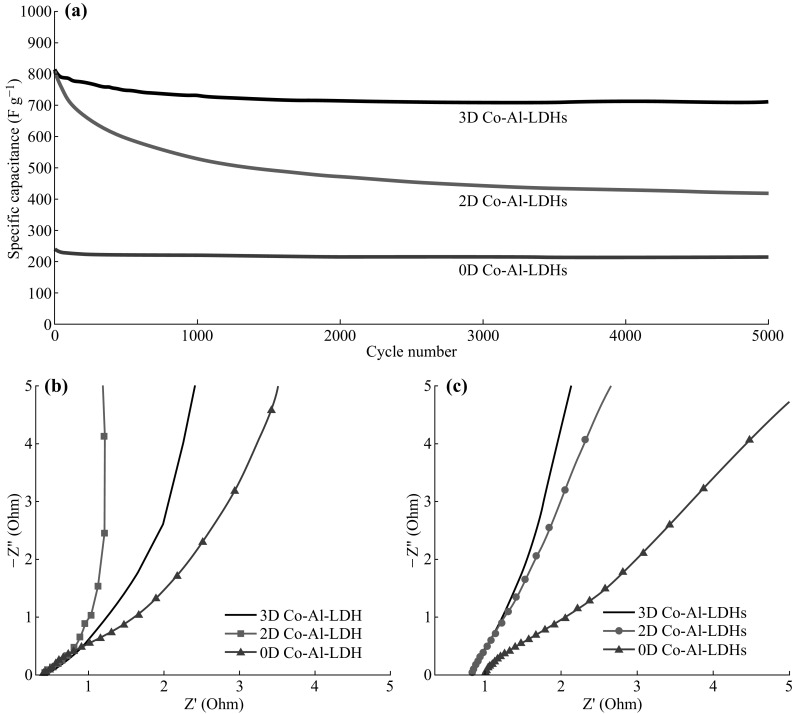
**a** Cycling performances at 5 A g^−1^ and EIS spectrums before **b** and after **c** 1000 cycles of all Co–Al-LDH samples

To further understand the effects of the unique structure on the electrochemical performance of 3D Co–Al-LDHs, the CV and galvanostatic charge–discharge curves of 2D Co–Al-LDHs and 0D Co–Al-LDHs were also determined, and the results are shown in Figs. S5 and S6. From Fig. S6, one can see that the 0D Co–Al-LDH nanoparticles had an initial capacitance of only ~250 F g^−1^, while 3D Co–Al-LDHs and 2D Co–Al-LDHs had initial capacitances of ~800 F g^−1^. The same initial capacitance of 3D and 2D Co–Al-LDHs is due to the similar 2D atomically thin structure. As shown in Fig. 5a, the capacitance of 2D Co–Al-LDHs decreases rapidly to 450 F g^−1^ (only 44% of the original value) after 5000 cycles at 5 A g^−1^ due to the re-stacking of 2D nanosheets, while 3D Co–Al-LDHs retains 93% of the initial specific capacitance (from 801 to 745 F g^−1^) and 0D Co–Al–LDHs retains almost 100%. The difference is related to the dispersion solution; that is, 2D Co–Al-LDHs were prepared in water, whereas 3D Co–Al-LDHs and 0D Co–Al-LDHs were prepared in mixed solvent or butyl alcohol. The surface of the 3D and 0D Co–Al-LDHs are modified by organic molecules, as supported by the FT-IR (Fig. 2b) and XPS (Fig. 3) spectra. The organic molecules adsorbed at the surface reduce the surface energy and improve the stability of the electrode materials [32–36]. EIS spectra of the electrodes were taken before and after the cycling process (Fig. 5b, c). High charge transfer and diffusion resistance were observed in 0D Co–Al-LDHs, which therefore led to poor capacitance. Nearly the same diffusion resistance was observed in 3D Co–Al-LDHs, whereas it became larger after the cycling process in 2D Co–Al-LDHs. The changes in diffusion resistance indicate that the 3D LDH structure can effectively prevent the re-stacking of 2D nanosheets, which further results in the unique cycling stability of the obtained 3D Co–Al-LDHs.

The high performance of the 3D Co–Al-LDH material can be ascribed to its unique 3D hierarchical structure. First, the atomically thin building units with thicknesses of approximately 1.6 nm (152 m^2^ g^−1^) can provide a large amount of electrochemically active sites to result in high capacitance. Additionally, the 3D hierarchical structures can prevent the re-stacking of nanosheets, and the surface modification of organic molecules can enhance the stability of 3D Co–Al-LDHs (Figs. S7, S8), leading to long-term cyclic stability. Furthermore, the pores in the 3D hierarchical structure are readily accessible for electrolyte, facilitating the transport of ions from the liquid to the active surface of the LDH. Finally, the 3D hierarchical structure can also supply more points to connect the conductive matrix in the electrode, which is beneficial to the conductivity and rate capability of the electrodes.

## Conclusion

A facile synthetic route was developed to directly prepare 3D hierarchical Co–Al-LDHs composed of atomically thin nanosheets. The as-obtained hierarchical Co–Al-LDHs show a high specific capacitance of 801 F g^−1^ at 5 A g^−1^, excellent rate performance with a capacitance of 677 F g^−1^ at 100 A g^−1^, and good cycling stability with only 5% decline after 20,000 cycles. Such excellent performance is derived from its atomically thin building units modified by organic molecules and its unique 3D hierarchical structure. This work may provide a promising alternative strategy to prepare other LDHs with enhanced electrochemical properties for supercapacitors.

## Electronic supplementary material

Below is the link to the electronic supplementary material.

Supplementary material 1 (PDF 1326 kb)
