# Supplementary material for: 3D Hierarchical Co–Al Layered Double Hydroxides with Long-Term Stabilities and High Rate Performances in Supercapacitors
Source: Nanomicro Lett. 2016 Dec 27;9(2):21. doi: 10.1007/s40820-016-0121-5 (PMC6223799; doi:10.1007/s40820-016-0121-5)
Supplement: Supplementary file 1 — Supplementary material 1 (PDF 1326 kb) [file 40820_2016_121_MOESM1_ESM.pdf]

Supporting Information for

3D Hierarchical Co-Al Layered Double Hydroxides with Long-term Stabilities and High Rate Performances in Supercapacitors

Jiantao Zai, Yuanyuan Liu, Xiaomin Li, Zifeng Ma, Rongrong Qi*, Xuefeng Qian*

Shanghai Electrochemical Energy Devices Research Center, School of Chemistry and Chemical Engineering and State Key Laboratory of Metal Matrix Composites, Shanghai Jiao Tong University, Shanghai, 200240, People's Republic of China

*Corresponding authors. E-mail address: rrqi@sjtu.edu.cn, xfqian@sjtu.edu.cn

Tel.: +86-21-54743262, Fax: +86-21-54741297

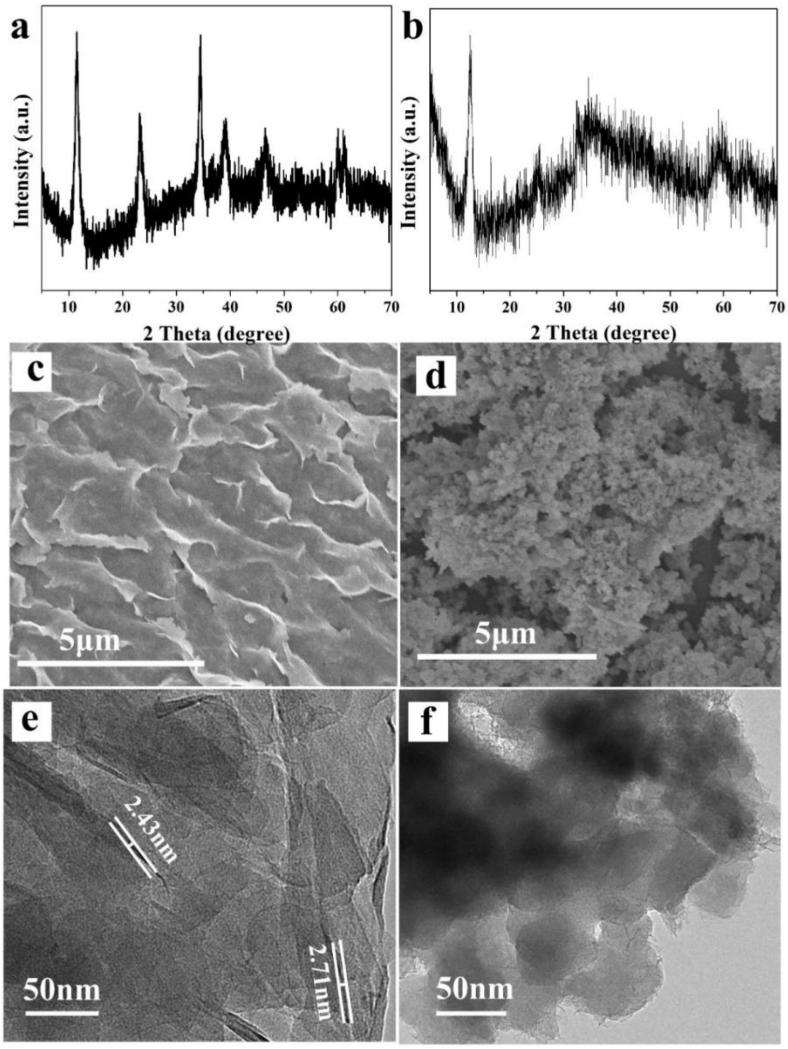

Fig. S1 XRD, SEM and TEM images of 2D Co-Al-LDHs (**a, c, e**) and 0D Co-Al-LDHs (**b, d, f**)

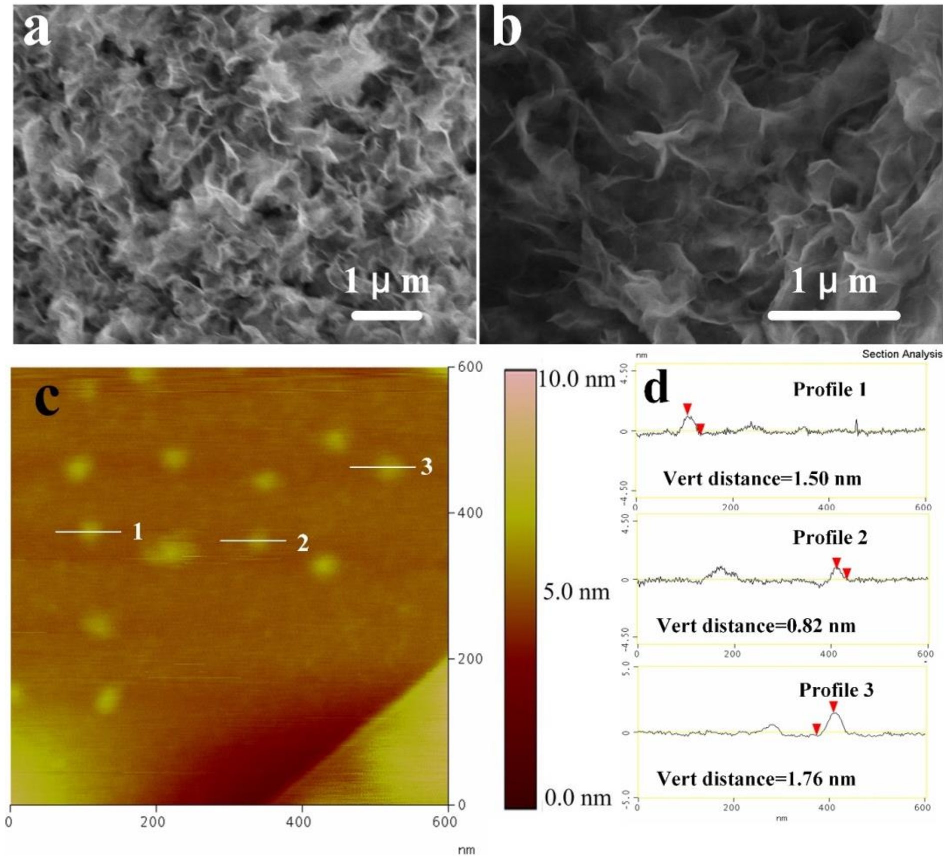

Fig. S2 SEM (**a-b**), AFM (**c**) and height profiles (**d**) images of atomically thin layered 3D Co-Al-LDHs. Profiles 1, 2 and 3 correspond to the numbered lines in a, respectively

To further determine the thickness of the as-prepared 3D Co-Al-LDHs nanosheets, samples dispersed on a mica wafer have been observed by Atomic Force Microscopy (AFM). The cross-sectional analysis corresponding to the highlighted profiles 1, 2 and 3 in the topography image clearly shows the average height of LDHs with less than 2 nm and smooth rounded shapes of ca. 54, 35, and 70 nm.

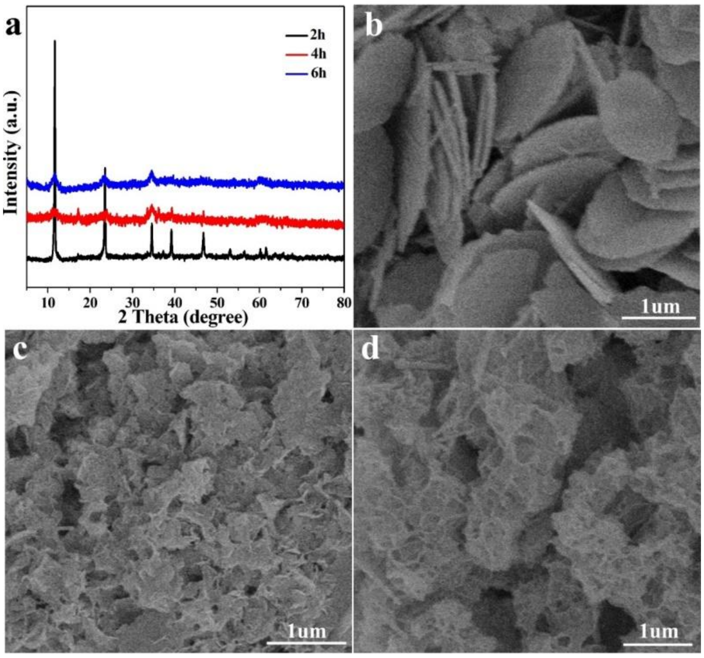

Fig. S3 (**a**) XRD of the target product Co-Al-LDHs (in 40 mL deionized water and 40 mL butyl alcohol) with different reaction time, and the corresponding SEM of 2 h (**b**), 4 h (**c**), 6 h (**d**)

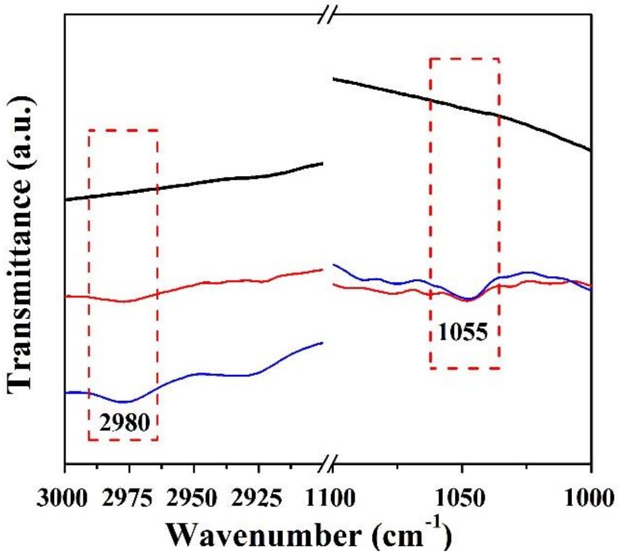

Fig. S4 Detailed FTIR spectra in the range of 3000-2900 cm^{-1} and 1100-1000 cm^{-1}

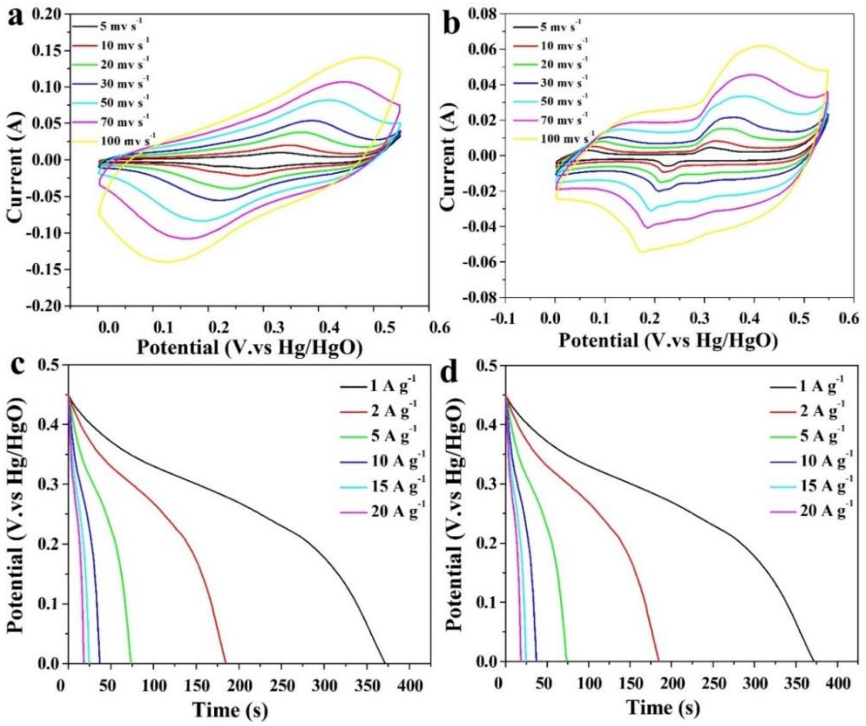

Fig. S5 CV curves of 2D Co-Al-LDHs (**a**), 0D Co-Al-LDHs (**b**) at different scan rates in 2 M KOH aqueous electrolyte, and galvanostatic charge–discharge curves at different current densities of 2D Co-Al-LDHs (**c**) and 0D Co-Al-LDHs (**d**)

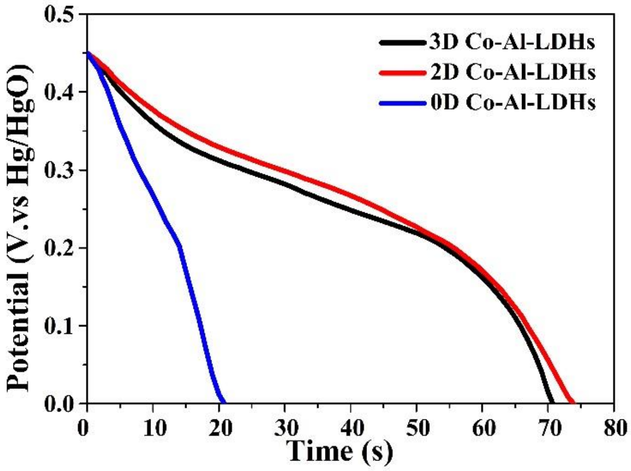

Fig. S6 Galvanostatic charge-discharge measurements of all LDHs at 5 A g^{-1}

Table S1 Comparison of the Csp, rate capability and cycle performances of our and the previous works

Electrodes based on materials	Csp ($F \cdot g^{-1}$)	Rate capability	cycle performances	Ref.
**GSP/Co-Al-LDH**	1043 (1 A g^{-1})	87% (20 A g^{-1})	3000 (88%, 100 mV s^{-1})	[1]
**Co-Al-LDH**	756 (1 A g^{-1})	44% (20 A g^{-1})	3000 (74%, 100 mV s^{-1})	
**Co-Al LDH/CNT**	884 (0.86 A g^{-1})	60% (4.3 A g^{-1})	2000 (88%, 1.72 A g^{-1})	[2]
**Co-Al LDH**	550 (0.86 A g^{-1})	51% (4.3 A g^{-1})	2000 (68%, 1.72 A g^{-1})	
**Co-Al LDH/GNS**	771 (1 A g^{-1})	73.2% (10 A g^{-1})	2000 (81%, 10 A g^{-1})	[3]
**Co-Al LDH**	641 (1 A g^{-1})	65.7% (10 A g^{-1})	2000 (64%, 10 A g^{-1})	
**Co-Al LDH/GO**	780 (1 A g^{-1})	80% (20 A g^{-1})	10000 (73%, 6 A g^{-1})	[4]
**Co-Al LDH**	701 (1 A g^{-1})	53% (20 A g^{-1})	10000 (52%, 6 A g^{-1})	
**Co-Al LDH/rGO**	621 (1 A g^{-1})	65% (8 A g^{-1})	5000 (92%, 4 A g^{-1})	[5]
**Co-Al LDH**	340 (2 A g^{-1})			
**LDH/Ni foil**	1031 (1 A g^{-1})	66% (100 A g^{-1})	2000 (92%, 4A g^{-1})	[6]
**3D Co-Al-LDHs**	838 (1 A g^{-1})	81% (100 A g^{-1})	20000 (95%, 5A g^{-1})	This work

GNS: graphene nanosheets
rGO: reduced graphene oxide
CNT: carbon nanotube
GSP: graphene sheets

Table S2 Specific surface area and total pore volume of different samples

Sample	3D Co-Al-LDHs	2D Co-Al-LDHs	0D Co-Al-LDHs
**Specific surface area (m^2 g^{-1})**	152 m^2 g^{-1}	90 m^2 g^{-1}	69 m^2 g^{-1}
**Total pore volume (cm^3 g^{-1})**	0.53 cm^3 g^{-1}	0.19 cm^3 g^{-1}	0.25 cm^3 g^{-1}

3D hierarchical Co-Al-LDHs are composed of atomically thin nanosheets, which is extremely thin (0.8-1.6 nm) than 2D Co-Al-LDHs (~3 nm). Thus, the surface area of 3D hierarchical Co-Al-LDHs twice of 2D Co-Al-LDH. While the 0D Co-Al-LDHs solid nanospheres with some nanosheets on their surface, and result the smallest surface area.

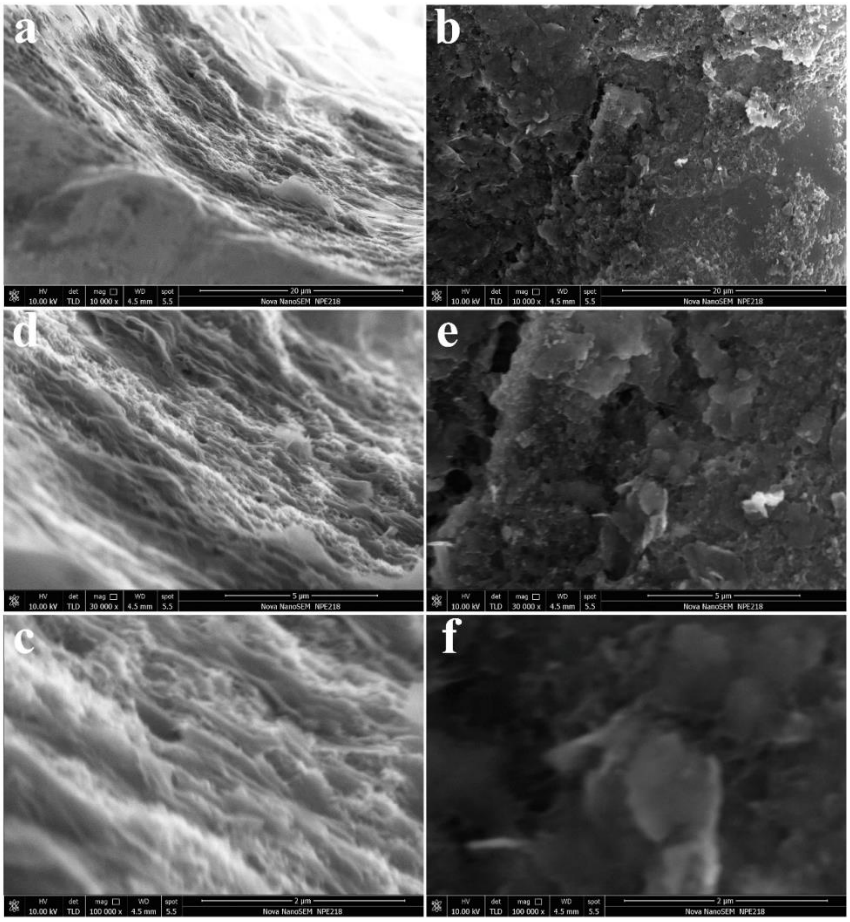

Fig. S7 SEM images of fresh (**a-c**) and cycled (**e-f**) electrodes of 2D Co-Al-LDHs

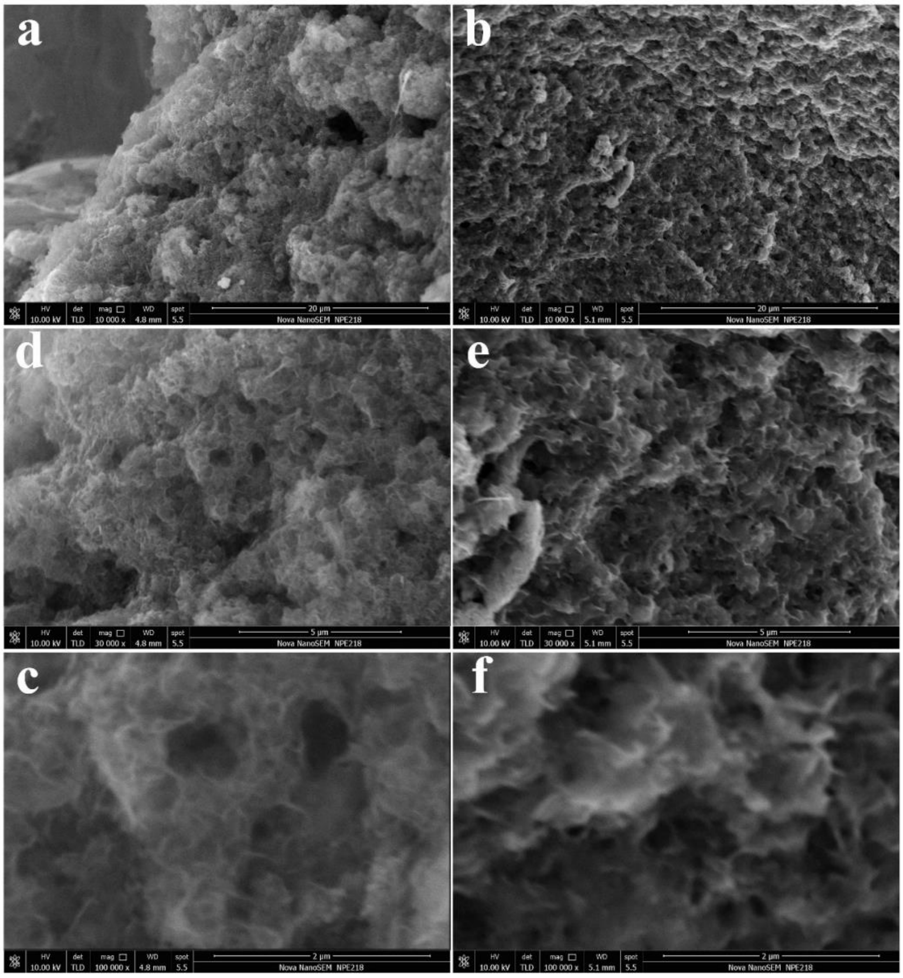

Fig. S8 SEM images of fresh (**a-c**) and cycled (**e-f**) electrodes of 3D Co-Al-LDHs

The fresh 2D Co-Al-LDHs electrode is composed of loosely stacking nanosheets, which is benefit for the diffusion of electrolyte. However, these nanosheets become tightly stacking nanoplates after cycling, which would be the reason of capacity fading. Different from the 2D Co-Al-LDHs nanosheets, no obvious morphology changes can be observed in the 3D Co-Al-LDHs electrode after cycling. This result further confirms that the hierarchical structures built by ultrathin nanosheets can effectively prevent the stacking of nanosheets and stabilize the overall structures, which is important to the cycling stability.

References

[1] X. Wu; L. Jiang, C. Long, T. Wei, Z. Fan, Dual support system ensuring porous Co–Al hydroxide nanosheets with ultrahigh rate performance and high energy density for supercapacitors. Adv. Funct. Mater. **25**(11), 1648-1655 (2015). doi:10.1002/adfm.201404142

[2] L. Yu, N. Shi, Q. Liu, J. Wang, B. Yang, B. Wang, H. Yan, Y. Sun, X. Jing, Facile synthesis of exfoliated Co–Al LDH–carbon nanotube composites with high performance as supercapacitor electrodes. Phys. Chem. Chem. Phys. **16**(33), 17936-17942 (2014). doi:10.1039/C4CP02020K

[3] L. Zhang, X. Zhang, L. Shen, B. Gao, L. Hao, X. Lu, F. Zhang, B. Ding, C. Yuan, Enhanced high-current capacitive behavior of graphene/CoAl-layered double hydroxide composites as electrode material for supercapacitors. J. Power Sources **199**, 395-401 (2012). doi:10.1016/j.jpowsour.2011.10.056

[4] J. Fang, M. Li, Q. Li, W. Zhang, Q. Shou, F. Liu, X. Zhang, J. Cheng, Microwave-assisted synthesis of CoAl-layered double hydroxide/graphene oxide composite and its application in supercapacitors. Electrochim. Acta **85**, 248-255 (2012). doi:10.1016/j.electacta.2012.08.078

[5] J. Hao, W. Yang, Z. Zhang, B. Lu, X. Ke, B. Zhang, J. Tang, Facile synthesis of three dimensional hierarchical Co–Al layered double hydroxides on graphene as high-performance materials for supercapacitor electrode. J. Colloid Interface Sci. **426**, 131-136 (2014). doi:10.1016/j.jcis.2014.04.003

[6] X. Liu, A. Zhou, T. Pan, Y. Dou, M. Shao, J. Han, M. Wei, Ultrahigh-rate-capability of a layered double hydroxide supercapacitor based on a self-generated electrolyte reservoir. J. Mater. Chem. A **4**(21), 8421-8427 (2016). doi:10.1039/C6TA02164F
